# The standard reconsolidation protocol for auditory fear-conditioning does not account for fear to the test context

**DOI:** 10.1371/journal.pone.0287193

**Published:** 2023-06-22

**Authors:** Jason W. Payne, Devon Merza, Dave G. Mumby

**Affiliations:** 1 Mississauga Department of Psychology, University of Toronto, Mississauga, Ontario, Canada; 2 Center for Studies in Behavioural Neurobiology, Department of Psychology, Concordia University, Montreal, Quebec, Canada; Federal University of Paraiba, BRAZIL

## Abstract

Research on memory reconsolidation has relied heavily on the use of Pavlovian auditory cued-fear conditioning. Here, an auditory cue (CS) is paired with a footshock (US) and the CS is later able to evoke a freezing response when presented alone. Some treatments, when administered to conditioned subjects immediately following a CS-alone (memory reactivation) trial, can attenuate the freezing they display on subsequent CS-alone (test) trials, in the absence of the treatment. This reduction in conditioned freezing is usually taken as evidence that the treatment disrupts post-reactivation reconsolidation of the memory trace representing the pairing of CS and US. We suggest an alternative interpretation that may account, either in whole or in part, for the attenuated freezing. The standard reconsolidation protocol (SRP) for auditory fear-conditioning has a design feature that results in second-order conditioning of fear to the test context, as this context is paired with the fear-evoking CS on the reactivation trial. Since freezing during the CS on the test will reflect the compound influence of contextual-fear and cued-fear, a post-reactivation treatment might attenuate freezing on the test by disrupting consolidation of second-order contextual-fear conditioning, even if it has little or no effect on the stability of the original cued-fear memory. This experiment confirmed that rats tested according to the SRP, in which the reactivation and test trials occur in the same context, freeze more on the test trial than rats that receive the reactivation and test trials in different contexts. This confound could lead to false-positive evidence of disrupted reconsolidation if it is not avoided or minimized, which can be accomplished with a modified protocol.

## Introduction

Auditory fear-conditioning is a popular technique for assessing memory reconsolidation in rodents. A footshock (US) is paired with an auditory condition stimulus (CS), and the CS subsequently elicits a fear response, which includes freezing. Some treatments administered to conditioned subjects immediately following a CS-alone trial can attenuate the freezing they show on subsequent CS-alone trials in the absence of the treatment [1]. When this reduction in conditioned freezing is absent in subjects that did not receive the CS-alone trial prior to treatment, it is typically concluded that the former treatment effect was due to disrupted post-reactivation reconsolidation of the memory trace representing the pairing of CS and US [2].

An alternative interpretation has yet to be ruled out for treatment effects on conditioned freezing in most reconsolidation studies using auditory fear-conditioning. Although, most of such studies follow the same standard reconsolidation protocol (SRP): a single conditioning trial is given in one context (Context A), followed 24-hrs later by a CS-alone trial (memory reactivation) in a second context (Context B), followed 24-hrs later by one or more CS-alone (test) trials in Context B [1]. This procedure has the potential to produce second-order conditioning [3–6] of fear to Context B, because this context is paired with the fear-evoking CS on the reactivation trial. Subjects that do not receive memory reactivation will not acquire fear of Context B. This difference between animals receiving “reactivation” versus “no-reactivation” could be problematic if context-fear influences the freezing displayed during CS presentation when animals are later returned to Context B for the test trial. Such compound effects of contextual and cued fear on the expression of fear-related behaviour should be expected when both contextual and cue stimuli are presented together, such as they are during CS presentation on the test trial of the SRP. Indeed, it has been shown that increasing background fear in mice accentuates freezing during presentation of a fear-evoking CS [7], and contextual fear accentuates other fear-related behaviour evoked by discrete stimuli in rats, such as the startle response [6]. If it is confirmed that the SRP produces second-order contextual-fear conditioning, this raises the possibility that attenuated freezing on the test trial following a post-reactivation treatment is not entirely due to disrupted reconsolidation of cued-fear memory, and that disrupted consolidation of context-fear learning, which occurs during the reactivation procedure also contributes, either in whole or in part, to the observed treatment effects.

The purpose of the experiment reported here was to provide some evidence for this theoretical position. Namely, whether or not the SRP for auditory fear conditioning produces second-order conditioning of fear to the test context. Second-order contextual fear conditioning has been demonstrated in experiments designed explicitly to produce it [6, 8], but in those studies there were multiple first-order and second-order conditioning trials [9]. The SRP involves only a single conditioning trial, and a single trial on which second-order conditioning might occur prior to the test trial. It was therefore important to determine the likelihood of higher-order conditioning resulting from the same procedures.

We mimicked the key features of the SRP (e.g., [1], and compared behaviour in rats treated accordingly with rats in two other conditions designed to control for the suspected contextual-learning confound. In accord with the SRP, adult male rats received a single conditioning trial in one chamber (Context A), followed 24-hrs later by a CS-alone trial (memory reactivation) in a different chamber (Context B), followed 24-hrs later by another CS-alone (test) trial in Context B. Here this is referred to as the SAME condition, because rats in this group received the reactivation and test trials in the same context. Their behaviour was compared to rats conditioned and tested in the DIFFERENT condition, which received the reactivation trial in a third context (Context C) and the test in Context B. The hypothesis was that rats conditioned and tested with the SRP would be more fearful of Context B on the test trial than rats treated with a procedure designed to avoid second-order fear conditioning to Context B. Specifically we predicted that rats re-exposed to the reactivation context on the test day would freeze more pre-CS than rats exposed to a different context.

## Materials and method

The subjects were male 43 Long-Evans rats, 14 weeks old and 6 male non-naive Sprague-Dawley rats approximately 52 weeks old (Charles River, St. Constant, Quebec). Rats were housed in polypropylene cages (48 × 25 × 20 cm) in a colony room maintained under a reverse 12:12 light-dark cycle (light onset at 8:00 p.m). The rats had continuous access to water and each received a daily ration of ~25 g of rat chow (Charles River Rodent Animal Diet, no. 5075) and the end of each day. The testing was conducted in two waves where 21 Long-Evens rats were part of the first wave and the remaining subjects run in the second wave. Available older non-naive rats but who were naive to fear conditioning, shock, the context chambers and rooms the context chambers were in were included a priori to marginally promote generalizability. These rats were divided equally among the two experimental conditions in the second wave. Adding heterogeneity to the sample reduces Type I error. In total there were 24 rats in Group SAME, and 25 rats in Group DIFFERENT. Three transparent conditioning chambers (28cm x 28cm x 25cm high) served as Contexts A, B, and C. The chambers were located in separate rooms, which differed in size, wall colours, lighting, ambient odours, and objects (e.g., furniture, fixtures, equipment) that were visible from within the chamber. Lighting was kept constant in each different experimental room with ordinary table lamps with 60-watt tungsten lightbulbs. Features were added to the outside of the chamber walls with coloured tape at different angles in order to further distinguish the contexts on the basis of proximal cues. Each chamber had a grid floor. Each chamber was counterbalanced to serve as Contexts A, B, and C. This counterbalancing was intended to further reduce the likelihood of Type I error. All animal procedures conformed to the guidelines of the Canadian Council for Animal Care. All procedures were approved by the Concordia University Animal Research Ethics Committee.

**Fig 1:**
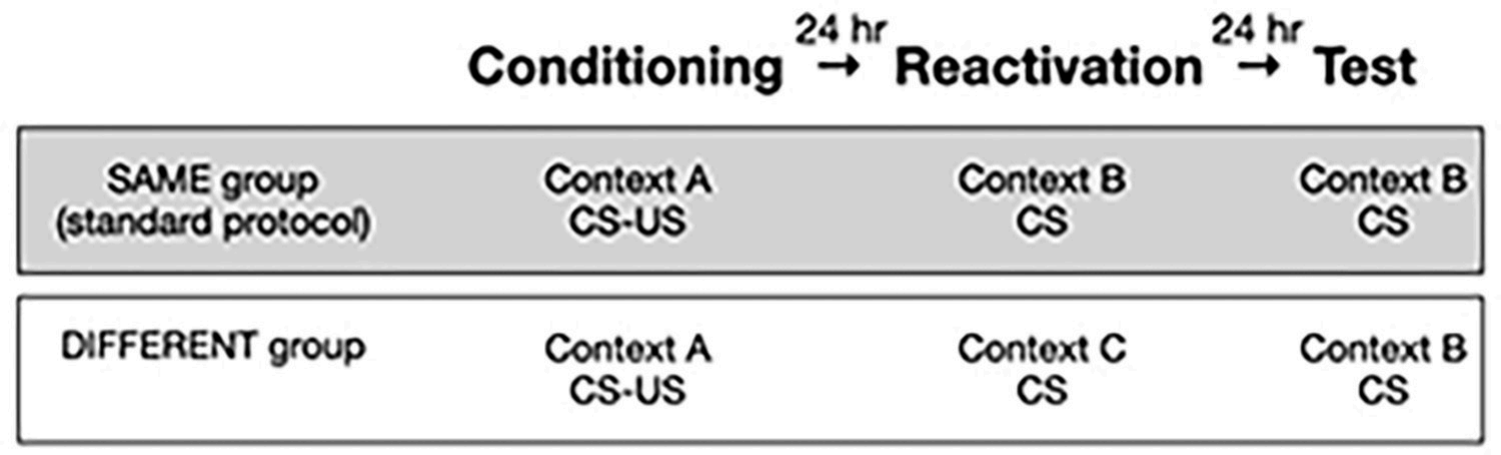
Experimental design.

The experimental design is illustrated in Fig 1. Consistent with the standard protocol, each trial lasted 300 seconds (s), and the inter-trial interval was 24 hours. The CS was a 30-second tone (2.9kHz, 75 dB), with an onset 150 seconds after the rat was placed in the chamber. The rat remained in the chamber for 120 seconds after CS offset. On the conditioning trial, a 1 second footshock US (2 mA) co-terminated with the CS. Freezing was quantified on the first trial (conditioning), the second trial (memory reactivation), and the third trial (memory test), separately for the 150-second pre-CS period, the 30-second CS, and the 120-second post-CS period.

## Results

**Fig 2:**
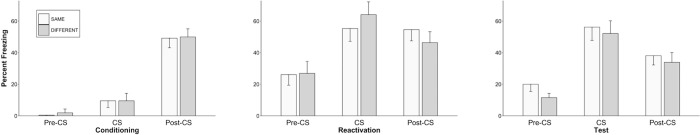
Percent time freezing. The Asterix represents a p-value < .05. Error bars represent one-tailed 90% confidence interval.

The percentage of time spent freezing during each period—pre-CS, during-CS, and post-CS—was scored for each animal. Between-group t-tests were used to compare freezing in Groups SAME and DIFFERENT for each period on each of the three. Consistent with our one-directional hypothesis, our statistical tests were one-tailed. The decision to use one-tailed t-tests was made prior to the data collection. None of the interpretations below would change if two-tailed t-tests were used with a standard statistical cut-offs of >.05. On the conditioning trial (Fig 2), there was very little freezing in either group during the pre-CS period or during the CS-presentation, but both groups showed a considerable amount of freezing following the shock (ie., during the post-CS period). On the reactivation trial (Fig 2), the groups displayed similar amounts of freezing during the pre-CS period, during the CS, and post-CS (all *p* > .05). Thus, the original fear-conditioning was equally effective in both groups, and they were well-matched in this respect. On the test trial (Fig 2), rats in the SAME group displayed significantly more (nearly double the) freezing during the pre-CS period than did rats in the DIFFERENT group (*t*(36.78) = 2.0396, *p* = .024, Cohen’s d = 0.59, Glass’s delta = 0.82, Hedges’ g = 0.59). This result remains statistically significant if a 2-tailed test is used as well as with a Welch t-test. The SAME group also spent nominally more time freezing than the DIFFERENT group during the CS (*t*(46.74) = 0.44, *p* = .332, Cohen’s d = 0.13, Glass’s delta = 0.13, Hedges’ g = 0.13) and post-CS (*t*(46.94) = 0.63, *p* = .265, Cohen’s d = 0.18, Glass’s delta = 0.17, Hedges’ g = 0.18) but those differences were not statistically significant (p > .05). Confidence intervals are reported on Fig 2. Alternatively, it could be argued that subjects should be removed if they did not demonstrate high CS-induced freezing of an outlier level of background freezing. Removing subjects did not affect our results meaningfully (i.e. only the test pre-CS period was statistically significant).

## Discussion

The results confirmed that pairing the fear-evoking auditory-CS with a specific context on the reactivation trial led to conditioning of fear to that context. Groups SAME was more fearful of the Context B on the test than was the DIFFERENT group. as the former groups displayed significantly more pre-CS freezing on the test trial. A likely explanation is that second-order contextual-fear conditioning occurred during the reactivation trial in the SAME group, but not in the DIFFERENT group, because the latter animals did not experience the CS paired with Context B on the reactivation trial. There is evidence of some generalization from the conditioning context, as all groups showed some pre-CS freezing when placed in a different context on the reactivation trial.

By convention, in order to determine whether a treatment has disrupted reconsolidation of fear-memory, at least two comparisons are made: 1) CS-induced freezing on the test trial is compared among groups that receive different levels of the treatment (e.g., drug versus vehicle) immediately after the reactivation trial. If subjects that receive a treatment after reactivation display less freezing during the CS on the test than subjects that receive a control treatment, it may be tentatively suspected that the treatment has disrupted reconsolidation of the conditioned fear-memory. 2) CS-induced freezing during the test must also be compared among subjects that did not receive a CS presentation and therefore did not have the conditioned fear-memory reactivated prior to treatment. If the treatment effect observed in subjects that received memory reactivation is absent in subjects that did not, the usual conclusion is that treatment effects observed in the former subjects reflect some influence on post-reactivation reconsolidation of the conditioned fear-memory. However, meeting these two criteria is not sufficient grounds for inferring a reconsolidation process. There must also be no confounded factors that provide a plausible alternative interpretation.

A potential complication in reconsolidation studies is the "new-learning" confound. It is self-evident that a procedure used to reactivate a memory also constitutes a learning episode. The concern is that new learning during a memory-reactivation procedure may later influence behaviour on memory tests for the original learning, and therefore, treatments given immediately after memory reactivation may exert their effects on subsequent memory tests by influencing consolidation of the new learning, rather than, or in addition to, reconsolidation of the original learning. Proponents of reconsolidation theory have addressed this concern by pointing out that the reactivation trial involves an extinction procedure, and if extinction has any effect on CS-induced freezing, it should be to reduce it; thus, disrupting the consolidation of extinction-learning should increase freezing. In fact, the opposite often occurs—subjects that receive an experimental treatment immediately after the reactivation trial show less freezing on the test than subjects that receive a control treatment—so, the typical results cannot be due to disrupted consolidation of new learning about the CS [2].

The CS, however, is not the only thing the subject experiences during the reactivation trial. The subject also experiences unique contextual cues, and it is possible that new learning related to the reactivation context might influence fear-related behaviour if the same context is used again for the test trial. This is a different “new-learning” confound from the one addressed previously by Nader and Hardt [2]. It has been shown previously that second-order contextual-fear conditioning can occur when a discrete cue is repeatedly paired with footshock in first-order conditioning, and later the same cue is paired with a new context in the absence of shock [8]. The present data are consistent with previous reports that rats display more conditioned freezing if they are trained and tested in the same context than if training and testing are done in different contexts [10]. The present data show that just a single pairing of a fear-evoking CS with a new context, which occurs with the SRP, is sufficient to condition a demonstrable degree of fear to that context.

It should be noted that no data were necessary to expose the potential for second-order contextual conditioning with the SRP. This potential is evident from an analysis of how that protocol is designed. The present findings elevate the likelihood the confound will influence the results of studies using this protocol from possible, based on the design analysis, to likely, based on the fact that we mimicked the SRP and saw substantial second-order conditioning. This represents more than just a potential nuisance—it could lead to false-positive findings of treatment effects on tests of cued-fear reconsolidation. The risk is high, because effective treatments are given immediately following the reactivation trial, which is when the newly acquired contextual fear would be undergoing cellular consolidation [2]. If a treatment disrupts consolidation of the contextual-fear conditioning, this could reduce freezing during the test trial, because the same contextual stimuli are present during the CS presentation, and there should be compound effects of cued-fear and contextual-fear on the conditioned response [11, but also see 9]. Such compound effects have been reported for conditioned-fear behaviour in mice [7], and in rats [6]. Thus, a treatment could potentially reduce the freezing displayed on later test trials, even if the treatment has no effect on the long-term stability of the original cued-fear memory.

As mentioned above, before inferring the involvement of a reconsolidation mechanism, any treatment effect observed in subjects that receive memory reactivation must be absent in subjects that do not receive reactivation. This pattern has been reported in several previous studies [1, 12–19], and it has consistently been interpreted as reflecting the existence of a cued-fear reconsolidation process. But, the lack of a treatment effect in a no-reactivation condition may be explained by the absence of second-order contextual-fear conditioning in those animals. Since they do not experience the fear-evoking CS in the reactivation context, the freezing they display during CS on the test will reflect mainly cued-fear (plus any non-associative fear, or contextual fear generalized from the conditioning context). Treatment effects in animals that experience memory reactivation may be absent in animals that do not experience reactivation prior to treatment simply because second-order contextual-fear conditioning occurs in the former but not the latter condition.

Second-order conditioning is intrinsically weaker than first-order conditioning [6], and it reasonable to question whether the second-order effect we observed was strong enough to account for the attenuation in freezing reported in previous studies that used the SRP. In the study by Nader and colleagues [1], for example, the differences in freezing during the test CS between anisomycin-treated rats that received memory reactivation and those that did not was greater than 40%. Pre-CS freezing on the test trial in the present experiments did not exceed 25%. If the compounded effects of contextual and cued fear on freezing are assumed to be merely additive or summative, then disruption of the consolidation of second-order contextual fear conditioning can account only partially for the reduced freezing in the anisomycin-treated rats of Nader and colleagues [1]. However, it is also possible that combining contextual and cued fear would produced a response less than sum of the two [9]. On the other hand, some phenomena, such as fear-potentiated startle, demonstrate that contextual or background fear amplifies or potentiates the cue-induced fear response [6]. Therefore, it is not enough to perform simple subtraction on freezing scores to determine the separate contributions of contextual-fear and cued-fear when both are occurring at the same time (as during CS presentation on the test trial of the SRP). The only way to rule out a contribution of contextual-fear to the overall freezing observed during CS presentation is to control for it within an experiment.

Several studies have used the SRP for auditory fear-conditioning [1, 12–19]. The occurrence and potential confounding influence of second-order contextual-fear conditioning cannot be ruled out from the data provided in most studies of cued-fear reconsolidation, because pre-CS freezing is not reported. Only freezing during the CS tends to be reported for the test trial. One exception is Cestari et and colleagues [12], reporting that mice spent nearly 50% of the pre-CS period freezing on the test trial. Thus generalized, non-associative fear cannot be ruled out as playing a role, however. The present findings highlight the importance of showing the data from all phases of a fear conditioning or test trial—pre-CS, during CS, and post-CS. The absence of those data in experiments using the SRP makes it impossible to estimate the extent to which either second-order conditioning or generalization from the conditioning context might have affected the results.

Future directions—as a helpful reviewers have pointed out—is that testing in a novel context could underestimate the amount of fear due to competition from exploratory behaviour. Future research should test this effect in phase 2, to exposing both groups to both B and C contexts. It would also be informative to include additional groups which were exposed to B or C but not given reactivation. This could quantify how much second-order conditioning of context B occurs after just one exposure to the context [see 5]. Lastly, future reconsolidation research should either test for this implication on anisomycin-treated rats or move forward with an A B C context experimental design to avoid the potential confound of contextual fear conditioning.

## Conclusions

Importantly, not all evidence for memory reconsolidation is questionable for the reasons we have given here. The present critique applies only to the standard protocol used to demonstrate reconsolidation of auditory cued-fear conditioning. There is much evidence of post-reactivation reconsolidation processes in contextual-fear conditioning, for example, and the standard protocol for demonstrating contextual-fear reconsolidation does not have the shortcomings of the SRP for cued-fear. Moreover, despite the flaw in the design of the SRP, this does not mean there is no evidence whatsoever of a post-reactivation re-stabilization period for cued-fear memories, because the findings in most studies that used the SRP are still logically consistent with the reconsolidation interpretation. It does mean, however, there is a plausible alternative interpretation based on disrupted consolidation that cannot be entirely ruled out. Definitive evidence of whether a treatment disrupts reconsolidation will depend on investigators adopting a modified protocol that uses different contexts for memory reactivation and test trials.
